# Profound Grief and Pulmonary Fibrosis – A Case Report

**DOI:** 10.1192/bjo.2025.10743

**Published:** 2025-06-20

**Authors:** Joyce Leong, Lay Ling Tan, Chiang Choo Goh

**Affiliations:** Changi General Hospital, Singapore, Singapore

## Abstract

**Aims:** Traditional Chinese Medicine (TCM) describes correlations between mental disorders, symptoms and physical organs, specifically identifying dominant emotions associated with specific organs. For example, in TCM sadness is correlated to the lung. TCM informs that psychological issues usually manifest as physiological dysfunction of the related organ. This encourages clinicians to consider the effects on various organs during management of mental disorders. This case study aims to explore the relationship and complexities between grief and idiopathic pulmonary fibrosis (IPF).

**Methods:** T was referred for grief counselling over her cats’ deaths and presented with respiratory symptoms which required further investigations. T is also a survivor of a horrific trauma 40 years ago, where an auntie wielded a knife at her and her mother. T’s mother died tragically while T bore scars across her arms, body, and the left side of her face visible till today. She was 6 years old. T reported that her father was deeply embittered, and never resolved his feelings of grief and anger. He died of lung fibrosis, which T attributes to his unresolved grief as she described how he would get breathless and could never talk about his late wife.

**Results:** Pulmonary fibrosis (PF) may be caused by many different things. IPF however is one type of PF where no cause can be identified.

Western literature concurs with TCM in that the link between disease and bereavement is strongest for the cardiovascular system. There are medical studies which investigated biological events that occur during the grieving process. They noted pathways through which grief might affect the immune system and increase vulnerability to physical illness.

T has no prior knowledge of TCM and no known family history of pulmonary fibrosis. However, T identified and believed that unresolved grief was a large contributor to her father’s lung condition.

**Conclusion:** Psychological issues as a potential risk factor to the development of lung diseases have not been studied in patients with IPF.

This case study highlights the importance of supporting T in her grief, if that may indeed reduce the probability of a lung pathology according to western literature and TCM.

A follow up study to explore existence of complex grief in a cohort of patients with IPF would shed light on the possible correlation between grief and lungs, as described by the TCM perspective.

